# Characterization of the 2017 Summer Heat Waves and Their Effects on the Population of an Area of Southern Italy

**DOI:** 10.3390/ijerph18030970

**Published:** 2021-01-22

**Authors:** Ernesto Infusino, Tommaso Caloiero, Francesco Fusto, Gianfranco Calderaro, Angelo Brutto, Giuseppe Tagarelli

**Affiliations:** 1 Department of Environmental Engineering (DIAm), University of Calabria, Via P. Bucci 41C, 87036 Rende, Italy; ernesto.infusino@unical.it; 2 National Research Council—Institute for Agricultural and Forest Systems in Mediterranean (CNR—ISAFOM), Via Cavour 4/6, 87036 Rende, Italy; giuseppe.tagarelli@isafom.cnr.it; 3 Multi-Risk Functional Center, Regional Agency for Environmental Protection of Calabria, Viale degli Angioini 143, 88100 Catanzaro, Italy; f.fusto@arpacal.it; 4 Health Protection Department of the Calabria Region, Viale Europa, Località Germaneto, 88100 Catanzaro, Italy; gianfranco.calderaro@regione.calabria.it (G.C.); angelo.brutto@regione.calabria.it (A.B.)

**Keywords:** temperature, humidity, humidex, hospital emergency department, Crati River, southern Italy

## Abstract

Knowledge of bioclimatic comfort is paramount for improving people’s quality of life. To this purpose, several studies related to climatic comfort/discomfort have been recently published. These studies mainly focus on the analysis of temperature and relative humidity, i.e., the main variables influencing the environmental stress in the human body. In this context, the present work aims to analyze the number of visits to the hospital emergency department made by the inhabitants of the Crati River valley (Calabria region, southern Italy) during the heat waves that accompanied the African anticyclone in the summer of 2017. The analysis of the bioclimatic comfort was performed using the humidity index. Results showed that greater the index, the higher the number of accesses to the emergency department, in particular by the most vulnerable population groups, such as children and the elderly.

## 1. Introduction

Bioclimatic comfort is a cognitive process resulting from different stimuli, in turn affected by various physical, physiological, and psychological factors [1]. Several studies on this subject underline how bioclimatic comfort is paramount to people’s life; therefore, knowledge of this process is extremely important. Heatwaves and high temperatures exceeding 36 °C can severely impact on bioclimatic comfort, and thus negatively affect people’s health, especially that of children, of the elderly and of those suffering from chronical illnesses [2,3]. Projections for the twenty-first century have predicted increasingly severe heat waves in those areas, e.g., southern Europe, where heat stress is a major influencing climatic factor [4,5,6]. Also, climate change and global warming are expected to significantly affect vulnerable areas such as the Mediterranean basin, with serious consequences [7]. In fact, heat can have several effects: increase in mortality [8], decrease in working/exercise capacity [9,10] and in cognitive performance [11,12]. Within this context, Ebi et al. [13] proposed an approach for assessing human health vulnerability and public health interventions to adapt to climate change. Alonso and Renard [14] studied the physiological and socio-economic vulnerabilities to heat waves in Lyon (France).

So far, the investigation of bioclimatic comfort has been carried out at different spatial scales focusing on different areas of the world (e.g., America [15,16,17,18], Europe [19,20,21], Asia [22,23,24] and Oceania [25]) and using many variables such as temperature, relative humidity of the air, wind and radiation. For example, Cetin [26] determined the bioclimatic comfort in Kastamonu City (Turkey) using temperature, relative humidity, and wind speed values. Patlakas et al. [27] studied the influence of low wind speed events, which are positively correlated to high-pressure systems and can consequently cause discomfort if associated with high temperatures. Among the several variables, temperature and the relative humidity of the air are key parameters in the study of climatic comfort/discomfort, because of their ability to alter the environment and cause bodily stress in the human population [1]. In fact, as Masterton and Richardson pointed out [28], people react differently to the same temperature but different humidity levels. For this reason, an index that aims at identifying the effects of heat on human health must include temperature and humidity [29].

Various indices exist to examine and evaluate the effects of heat on human health. These indices could be calculated through empirical formulas, depending on specific environmental parameters such as temperature, relative humidity, solar radiation and wind speed. Cannistraro et al. [30] produced a thorough description of some of the main indices (e.g., Scharlau Index, Heat Index, New Summer Simmer Index, Equivalent Temperature, Thom index and Wind Chill) evidencing for each index the parameters considered and the field of application. For example, the Summer Simmer Index considers temperature and relative humidity and can be applied for temperature ranging between 22 °C and 53 °C. Temperature, relative humidity, solar radiation and wind speed are required to determine the Equivalent Temperature where the parameters depend on the geographical location. Finally, for the calculation of the Wind Chill temperature (<11 °C) and wind speed (2 ÷ 24 m/s) data must be considered.

These and other indices have been largely applied in literature. For example, the Heat Index has been used to assess the variability of heat exposure in Knoxville, Tennessee (USA) considering urban and downtown stations [31]. Results suggested that trees can be associated with greater humidity and, thus, with higher Heat Index values. Moreover, in the same city, lifestyle surveys have been conducted on residents during a heatwave to understand heat exposure [32]. Always in the United States, heat-vulnerable populations and regions in New York State have been identified through the heat vulnerability index [33]. Europe’s thermal bioclimate for the summer season has been assessed through the Universal Thermal Climate Index (UTCI) maps by Di Napoli et al. [34] using data from the European Centre for Medium-Range Weather Forecasts (ECMWF) ERA-Interim reanalysis. Then, in the same paper, thermal bioclimatic data have been correlated with mortality data in 17 European countries evidencing an increase in mortality for moderate and strong UTCI classes.

The humidity index (Humidex) is one of the most popular indices, and consists of a simple thermal comfort index using air temperature and humidity [28]. This index can be applied for temperature values ranging between 20 °C and 55 °C [30]. Its results directly compare with dry temperature in degrees Celsius and its values have corresponding degrees of thermal comfort. For these reasons, the index is highly comprehensible. In addition, Humidex is more easily calculated than other, more complex indices (e.g., the Universal Thermal Climate Index or the Physiological Equivalent Temperature). Indeed, only two input parameters are required (temperature and humidity), which meteorological stations commonly measure, since they are extremely significant for a variety of enquiries in almost every branch of atmospheric science [35]. For these reasons, several studies have extensively applied Humidex. Błażejczyk and Twardosz [36] detected significant changes in bioclimatic conditions in Cracow (Poland) during the 1826–2006 period, mainly in the winter season. In Canada, Mekis et al. [37] used Humidex and wind chill indices to analyze changes in extreme heat and extreme cold events for the period 1953–2012 at 126 climatological stations, showing a significant increase in both the variables. Humidex was also applied to evaluate the effects of the urban heat island on Hradec Králové (Czech Republic) city residents and visitors [38]. Giannopoulou et al. [39] studied human thermal comfort in the Greater Athens area (Greece) during June–August 2009 showing that the urbanization of Athens has dramatically affected outdoor thermal comfort conditions. Oleson et al. [40] investigated how urbanization, heat stress, and climate change interact over the U.S. and southern Canada. In particular, they implemented five indices of heat stress in a model and detected higher urban-rural heat stress with Humidex rather than using temperature alone.

The use of this kind of indices is particularly important especially in the development of specific alert systems for heat waves, which in the future will lead to a higher number of fatalities and diseases in the most vulnerable population in terms of age, clinical situation and socio-economic conditions, especially in large urban areas [41]. For example, it has been estimated that the 2003 summer heat wave in Europe caused over 40,000 deaths. In particular, a mortality equal to 3134 in 21 major cities of the Italian regions has been observed in the period 1 June–31 August 2003 when compared to summer 2002 [42]. In fact, during heat waves there is an increase in hospital admissions, and heat waves longer than five days can cause increases in mortality 2–5 times higher than those of shorter duration [43]. It has also been observed that early heat waves, at the beginning of the summer season, have a greater impact on the population’s health than episodes of equal intensity later in the season [44].

Following Meehl [45] who defined a heat wave, or heatwave, as a period of excessively hot weather, which may be accompanied by high humidity, the aim of this paper is to assess the direct impact of the 2017 heat waves on the health of the population of the Crati River valley. With this aim, the Humidex and the admissions to the hospital emergency department during the heat waves of summer 2017 have been studied. Among the several indices, the Humidex has been selected because it requires only temperature and humidity data, which are both available in the study area. Moreover, as regards the population analyzed, it is especially suitable to this study because it lives in a very interesting area for its territorial, climatic and socio-economic peculiarities. The territory, in fact, has a mainly flat conformation and it is crossed by the longest river in Calabria. Moreover, Sirangelo et al. [46] evidenced the different influence of temperature and relative humidity on the Humidex behavior. Finally, in the Crati River valley is Cosenza, the fifth Calabrian city by number of inhabitants, which hosts one of the four Hospital Hubs, able to provide specialist diagnostic and therapeutic services, of the Calabria Region.

## 2. Materials and Methods

### 2.1. The Humidity Index: Humidex

Humidex (*H*) is an index that combines temperature and humidity to better describe the effects of heat on living organisms. The index is expressed by the empirical equation:

(1) $$H=T+\frac{5}{9}\left(e-10\right),$$

where *T* is the air temperature (°C), and *e* is the partial vapor pressure (hPa) [28].

As this last variable is not easily available, it can be evaluated by using the relative humidity, *U_r_* (%), and the saturation vapor pressure, *e_sat_* (hPa), through the relationship:

(2) $$e=U_{r}\cdot e_{sat},$$

where *e_sat_* depends on the air temperature alone, and can be calculated through Tetens’ formula [47]:

(3) $$e_{sat}=6.112\cdot 10^{\frac{7.5\cdot T}{T+237.7}},$$

Humidex has no specific measurement unit; anyway, it can be associated to the same unit of the temperature (°C), though it is not a physical variable.

As a result, the temperature perceived by the human body can be easily found by using the observed values of temperature and relative humidity in Equations (1)–(3), and detecting the discomfort level corresponding to the Humidex value (Table 1).

### 2.2. Study Area and Meteorological Data

**Figure 1:**
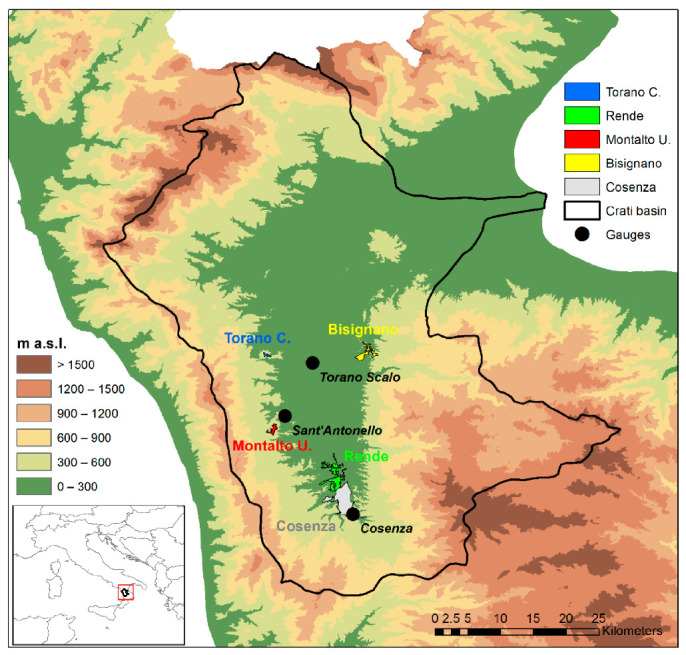
Location of the Crati River basin, of the main municipalities and of the selected gauges on a Digital Elevation Model (DEM).

The area under investigation is the valley of the Crati River, the main river of the Calabria region, which is located at the toe of the Italian peninsula, in the center of the Mediterranean basin (between 37°54′ to 40°10′ N and 15°36′ to 17°13′ E). The Crati River basin has an area of 2447.7 km^2^ and a perimeter of about 320 km (Figure 1). The basin is characterized by a typical Mediterranean climate, presenting sharp contrasts due to its position within the Mediterranean Sea and its orography [48]. The Köppen-Geiger classification [49] identifies the climate of the study area as a hot-summer Mediterranean climate. In fact, the study area falls within the Calabria region, whose climate is characterized by mild winters and hot summers with low precipitation. In particular, the Ionian side is influenced by warm air currents coming from Africa, and high temperatures with short and heavy rainfalls. Western air currents influence the Tyrrhenian side, which presents milder temperatures and mainly orographic precipitation. In the inland zones, colder winters with snow and cooler summer with some precipitation are observed [50].

**Figure 2:**
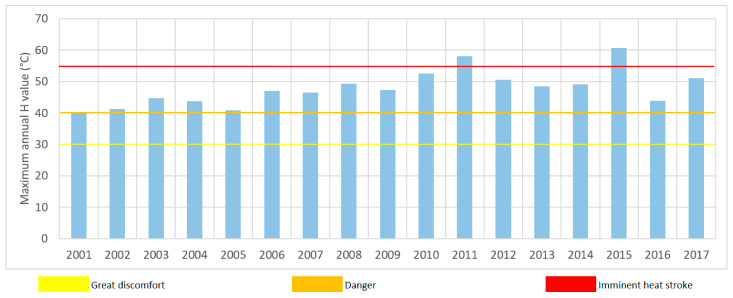
Maximum annual value of the Humidex in the Cosenza gauge.

The meteorological database used in this study consists of hourly temperature and humidity series registered in three stations in which both data are available: S. Antonello of Montalto Uffugo, managed by the GICA laboratory, the Department of Environmental (DIAm) of the University of Calabria, and Cosenza and Torano Scalo managed by the Multi-Risk Functional Centre of the Regional Agency for Environmental Protection (Table 2). These stations have also been selected because they are representative of different conditions. In fact, the Cosenza station is positioned close to a river in an urban area, the S. Antonello station is placed in an open area without buildings and far from the river, and the Torano Scalo station is situated in a rural, little urbanized area near to the river. Figure 2 shows the main features of the stations.

In order to evaluate the possible existence of temporal tendencies in extreme heat events, characterized by stagnant warm air masses and consecutive nights with high minimum temperatures [51], temperature values were analyzed for trends. Their statistical significance was assessed with the Mann-Kendall nonparametric test [52,53].

### 2.3. Hospital Emergency Department Data

The present study is a retrospective observational study based on the database of the Calabria Region’s Health Protection Department, which manages the data from the several hospitals of the Calabria region. Specifically, in the present study, the Emergency Department (ED) data of the Annunziata Hospital of Cosenza over the period January–-December 2017 have been considered.

The database consists of 66,798 visits. According to privacy rules, for each patient and in anonymous form, the following information was collected: age; gender; year, day, hour and minute of access; municipality of residence; mode of hospital discharge (sent back home, refused admission, removal, and hospitalized); the symptoms reported by the patients and the triage access code (white, green, yellow, and red).

Due to the large number of data and to the presence of several diagnostic data in the fields, specific inclusion and exclusion criteria have been used to identify the cases to be included in the study. Specifically, in order to assess the direct impact of the 2017 heatwaves on the health of the population of the Crati River valley, first only patients living in the main municipalities of the study area (i.e., Cosenza, Rende, Montalto Uffugo, Bisignano and Torano Castello) have been selected (Figure 1). Then, only patients with the presence of symptoms that can be clinically related with a heatwave have been considered, therefore, abdominal and thoracic aches, fever, altered heart rate, hypertension and other cardio-vascular ailments, have been included in the study. Patients affected by neurological, dermatological, gynecologic, urological, ophthalmic ailments or with traumas, poisoning, and allergic reactions, have been excluded. As a result, 29,031 visits have been finally considered for the study (Table 3). In order to allow an in-depth analysis of the data, in this paper two simple indexes have been evaluated.

The first index *A* (%) represents the incidence on the population of the monthly visits to the emergency department:

(4) $$A=\frac{\mathrm{MV}*100}{P}$$

with MV indicating the total number of monthly visit and P the population.

Moreover, in order to evidence daily visits higher than average values, a daily visits index *I_a_* has been simply evaluated as follows:

(5) $$I_{a}=\frac{DV}{DV_{a}}$$

where *DV* is the number of daily visits in the ED and *DV_a_* is the mean annual number of daily visits in the emergency department. Obviously, *I_a_* values higher than 1 means a number of visits higher than average and vice versa.

## 3. Results

Several studies indicated the different influence of temperature and relative humidity on the Humidex behavior, showing that the discomfort conditions are significantly related to air temperature, while the impacts of humidity are of less importance [46]. Within this context, a preliminary analysis focused only on temperature trend data in the period 2001–2017. Although past studies conducted in the Calabria region showed a growing risk of extreme heat events [54,55,56], results of this preliminary analysis did not show a statistically significant temperature increase in the period 2001–2017. In any case, 2017 has been identified in all the stations as the second hottest year in the period 2001–2017. As regards relative humidity, from 2011 to 2015 the highest values have been detected. In the Appendix A, the hourly temperature and relative humidity values registered in the Cosenza gauge during the study period 2001–2017 are shown.

After the preliminary analysis of the temperature data, Humidex has been evaluated at hourly time scale. As a result, for all the stations, an increase in the Humidex values has been detected during the observation period, with the highest values occurring after 2006, when the maximum annual values always exceed the value 46, with the exception of the year 2016 (Figure 2).

In particular, in 2011, in 2015, and in 2017 more than 150, more than 200 and about 100 time intervals showed Humidex values higher than 40, respectively. Moreover, in these years, the “Imminent Heat Stroke” threshold was exceeded, thus showing high human risks due to heat exposure.

**Figure 3:**
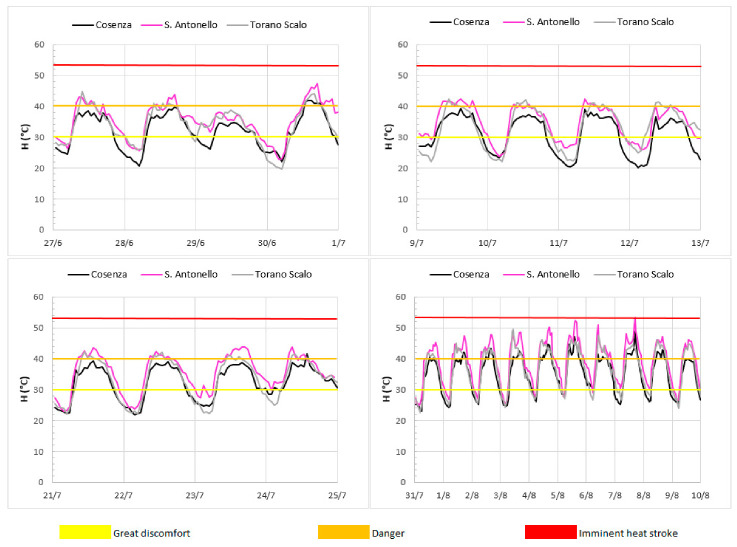
Temporal evolution of the Humidex (°C) during the four heat waves of summer 2017 (27 June–1 July; 9 July–13 July; 21 July–25 July and 31 July–10 August).

The year 2017 has not been identified as the year with the highest Humidex values, probably due to the lower humidity values than the previous years; in any case, this year is particularly important because in the period June-August the Crati River valley was affected by four different heat waves. Humidex temporal evolution during the summer (from June to August) of 2017 is shown in Figure 3. As a result, in this period, the index values exceed the “Danger” threshold in 21 days, with the longest exceeding sequence identified between the end of July and the beginning of August (10 consecutive days). Moreover, during this period, all the events have been characterized by tropical nights with minimum temperatures higher than 20 °C.

**Figure 4:**
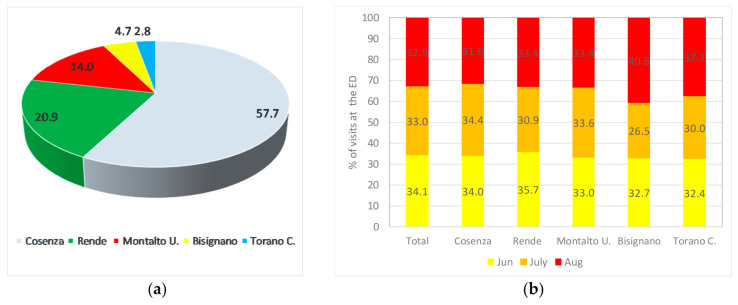
(**a**) Percentage of access to the ED for the main municipalities of the Crati River valley; (**b**) percentage of access in the summer months of 2017.

With the aim to assess the direct impact of the 2017 heatwaves on the health of the population of the Crati River valley, Emergency Department (ED) data of the Annunziata Hospital of Cosenza have been thoroughly analysed. First, in order to have an idea of the annual ED data, a total of 29,031 ED visits between January–December 2017 have been extracted (Table 3). The majority of the visits (57.7%) belong to the municipalities of Cosenza (Figure 4a), followed by Rende (20.9%) and Montalto (14.0%). Then, taking into account the higher influence of temperature than humidity on the Humidex evaluation, and considering that the study area is characterized by a Mediterranean climate, the ED data from June to August, which could be related to heat waves symptoms, have been considered in the following analyses. Figure 4b reports the access percentage in the summer months.

Generally, in summer 2017 the majority of the accesses has been detected in June (34.1%), followed by July (33.0%) and August (32.9%). This general behavior varies among the different municipalities. In fact, for Bisignano and Torano C. the maximum number of summer accesses has been registered in August (40.8% and 37.7%, respectively), while for Cosenza and Montalto U. the maximum number of summer accesses has been registered in July (34.4% and 33.6%, respectively). The differing temporal trends in ED visits between locations could be explained considering that, in this period of the year, there is a continuous mobility of people from inland to coastal areas.

**Figure 5:**
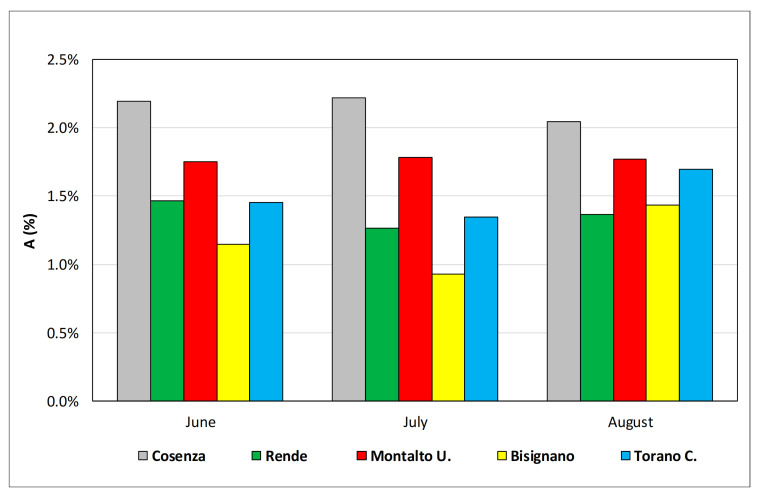
Incidence on the population of the monthly visits to the ED.

As regards the incidence on the population of the monthly visits to the emergency department (Figure 5), Cosenza is the municipality with the highest access percentage, with more than 2% of the population accessing the ED in the summer period, possibly due to the proximity of the Annunziata Hospital, the only one in the considered municipalities. Conversely, in Bisignano the farthest municipality from the ED, the number of accesses is the lowest, reaching a maximum value of about 1.4% in August.

**Figure 6:**
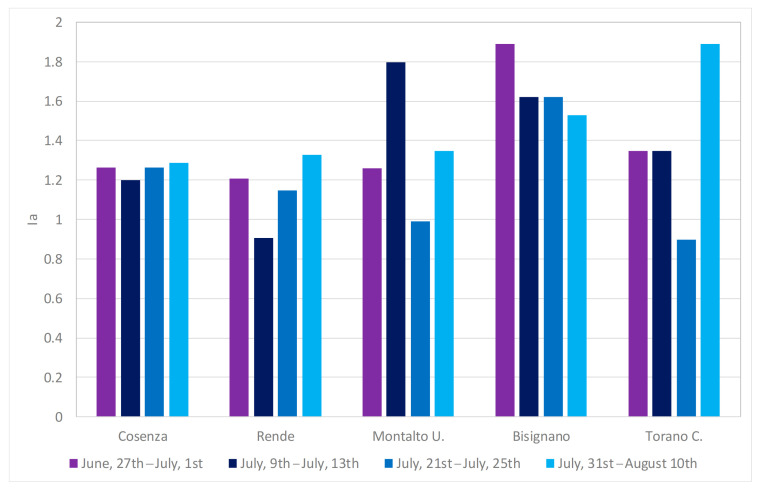
*I_a_* values for the different municipalities in the four heat waves of summer 2017.

In order to highlight daily visits higher than average values, for each event and for each municipality, the daily visits index has been evaluated (Figure 6). Results show daily accesses at the ED always higher than average, with the exception of the municipality of Rende in the second event and of the municipalities of Montalto U. and Torano C. in the third event. The highest number of accesses have been registered in Bisignano for the first and the third events, with *I_a_* values higher than 1.6, in Montalto U. for the second event (*I_a_* about 1.8) and in Torano C. for the fourth event, with *I_a_* equal to 1.89.

**Figure 7:**
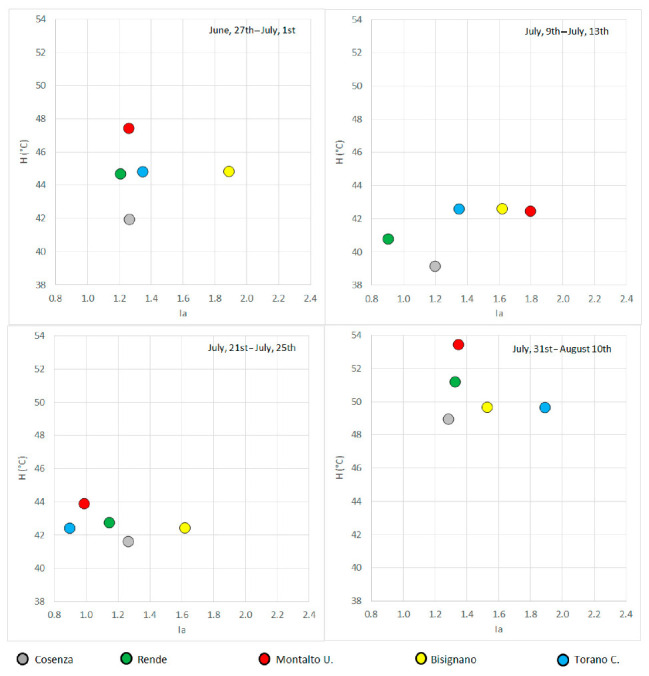
Comparison between *I_a_* and *H* values in the different heat waves of 2017.

For each municipality and for each heat wave, the *I_a_* values have been compared with the Humidex values (Figure 7).

In order to perform this comparison, due to proximity and to temperature characteristics, the *H* values for Rende have been evaluated as an average of the *H* values estimated in Cosenza and Sant’Antonello, while the *H* values for Torano have been also used for Bisignano. Results evidenced that high *I_a_* values have been identified in the fourth event, when the high *H* values (ranging between 48.9 and 53.4) have also been identified. In particular, in the fourth event the highest *I_a_* values have been detected in the smaller municipalities (i.e., Torano C. and Bisignano) with respect to Cosenza, although with similar *H* values, probably because of a lower presence of air conditioning systems and a greater incidence of outdoor works. Moreover, in Bisignano in the first event, higher *I_a_* values than in the fourth have been identified, despite the latter presented a higher Humidex. This particular behavior could be due to the traditional medieval horse tournament, the so-called “Palio”, which induces several emigrants to return in the last week of June, with a consequent large increase in the resident population.

**Figure 8:**
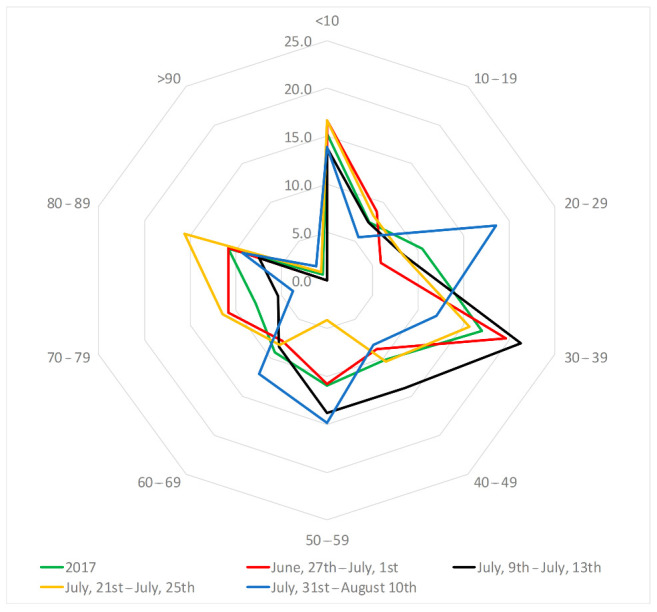
Incidence of accesses to the ED of the Annunziata Hospital of Cosenza for different classes of age in the four heat waves and during the whole summer 2017.

In order to detect the most exposed categories, considering age and gender, the total number of accesses to the ED during the four heat waves have been grouped in several classes. In particular, as regards age, 10 classes have been considered and the incidence of access for the different classes has been evaluated (Figure 8). For the whole summer 2017, results indicated a higher incidence of access for children in the age group up to 9 years, and for the elderly in the age group between 80–89 years, who can generally be considered the groups most at risk, but also for the group between 30–39 years. This general behavior has also been confirmed for the four events, with the exception of the last one in which a high incidence of accesses in the group between 20–29 has also been identified, probably because people in this age range are more active than the others. On the contrary, for all the events the lowest ED accesses have been identified in the group >90 years. Moreover, a low incidence of access has been identified in the third event, for the age group between 50–59 years, and in the fourth event for the age groups between 10–19 years and 70–99 years.

**Figure 9:**
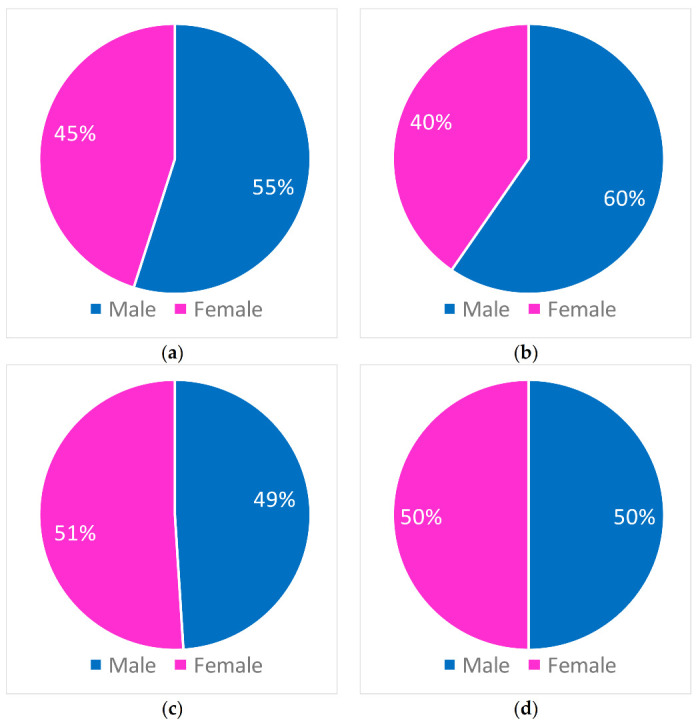
Incidence of accesses to the ED of the Annunziata Hospital of Cosenza for different sex in (**a**) 27 June–1 July (**b**) 9 July–13 July (**c**) 21 July–25 July (**d**) 31 July–10 August.

Figure 9 shows the percentage of accesses for different sexes in the four heat waves. Results indicated that heat waves affected both male and female subgroups. In particular, in the first and in the second event a higher percentage of males accessed the ED of the Annunziata Hospital of Cosenza, while in the third and in the fourth event a similar percentage has been identified.

## 4. Discussion

Emergency admissions to the Annunziata Hospital of Cosenza resulted especially high during summer 2017, when in 21 days the Humidex values exceeded the “Danger” threshold, with minimum temperatures higher than 20 °C at night. In addition to total admissions, a significant number of ED admissions were generally detected for some categories such as male and age between 0–9 and between 80–89. The overall increase in access during summer 2017 is consistent with other research on this topic [57,58,59,60,61], although some researchers pointed out different results [62].

As regards age, results of this study agree with the ones provided by some previous research, which showed a greater impact of heat waves among the elderly and young children [63,64,65], although other studies showed elevated accesses in the ED for all age groups [66,67,68,69]. In fact, elderly and young children fall within population subgroups characterized by a limited capacity of physiological thermoregulation or reduced possibility of implementing protective behaviors. Moreover, in this study a higher incidence of access has also been detected for the age groups between 20–29 years and between 30–39 years, thus confirming the results obtained by Davis and Novicoff [70]. The number of people in the highest age group (greater than 70 years) accessing the ED of the Annunziata Hospital in 2017 seemed to be not as high as expected but, as demonstrated by Davis and Novicoff [70], this can be mainly due to other factors, such as isolation, lack of access to immediate care, and people‘s perception that they are not in danger.

Considering the symptoms reported by the patients, some people accessing the ED have been classified with the codes related to cardiovascular diseases (Abdominal pain, Thoracic pain and Dyspnea). In fact, past studies evidenced a relationship between cardiovascular diseases and accesses to the ED (e.g., [71]), even though opposite results have been detected in other studies [72,73]. The majority of the people who accessed the ED during summer 2017 have been classified with the code indicating “Other Conditions”. In fact, during heat waves generally several patients show mild heat-stress illness symptoms such as edema, syncope, cramps, dehydration, and heat exhaustion [70]. These results confirm heat-related cardiovascular diseases detected in other studies [74,75], but also suggest that heat waves have an impact on several diseases [76].

Finally, results of this study also indicate that heat waves affect both male and female subgroups, thus confirming the literature showing inconsistency regarding differences in gender responses to heat [70]. In fact, while some differences by sex have been detected in some studies [77], others did not evidence any difference [78,79].

In order to properly contextualize the results of this study, some important final remarks must be made concerning the limitations of this study. First, this analysis has been performed using data for a single hospital and for one year only, so the extent to which these findings are generalizable is uncertain. Then, as pointed out by Davis and Novicoff [70] it is possible that other factors not incorporated in this study, such as socioeconomic status and pre-existing conditions, also affect ED accesses. Unfortunately, patient-level details were not available in this research, so these components were beyond the scope of this study. Finally, the large use of predefined diagnostic categories may potentially mask the real relationship between heat waves and accesses to the ED.

## 5. Conclusions

The comprehensive meteorological index Humidex was used to analyze the combined effects of temperature and relative humidity on people living in the Crati River valley (south Italy), a very interesting area for its territorial, climatic and socio-economic peculiarities. Daily emergency department admissions from five municipalities close to the Annunziata Hospital of Cosenza over the period January–December 2017 have been examined to determine if admissions were higher during the summer heat waves. As a result, in summer 2017, the Humidex values exceeded the “Danger” threshold in 21 days. In these days, daily accesses at the ED emerged to be always higher than average for each considered municipality. Results also evidenced that the highest accesses have been identified in the fourth event, when the high Humidex values have also been identified. Moreover, our results show that the increase in temperature and relative humidity could greatly exacerbate the risk of morbidity effects that extend across the cardio-vascular diseases spectrum, mostly for children in the age group up to 9 years and for the elderly in the age group between 80–89 years. Finally, an inconsistency regarding differences in gender responses to heat has been evidenced.

Studies like this one may assist relevant government agencies in the development of more accurate heat alerts, and in the provision of measures to prevent or reduce temperature-related health hazards. However, also in the light of increasingly extreme atmospheric conditions due to climate change, further investigations are required to explore the multidimensional aspects of the associations between heat wave exposure and ED visits.

## Figures and Tables

**Table 1:** Classes of the Humidex index and linked discomfort levels [47].

Humidex	Discomfort Levels
*H* < 29	Discomfort perceived by a few people
30 < *H* < 34	More or less significant malaise
35 < *H* < 39	Quite intense malaise. Caution. Limit some heavy physical activities
40 < *H* < 45	Sense of general malaise. Danger. Avoid efforts
46 < *H* < 53	Serious danger. Suspend physical activities
*H* > 54	Impending heatstroke (danger of death)

**Table 2:** Main features of the selected stations.

Name	Longitude	Latitude	Elevation	Measurement	Period
Cosenza	16.265	39.286	242	P, T, U, R ^1^	2001–2017
S. Antonello	16.172	39.421	300	P, T, U ^1^	1997–2017
Torano Scalo	16.209	39.493	97	P, T, U ^1^	2001–2017

^1^ P = Rainfall, T = Temperature, U = Humidity, R = Radiation.

**Table 3:** Municipalities, distance from the Emergency Department of Annunziata Hospital of Cosenza, population and visits from January to December 2017 of the analysed sample.

Municipality	Context	Distance to Hospital (km)	Population	Visits to ED Annunziata Hospital	Annual Visits (%)	Daily Visits (%)
Cosenza	Urban	0	67,239	16,753	24.92%	0.0683%
Rende	Urban	10	35,727	6054	16.95%	0.0464%
Montalto U.	Rural	20	20,213	4060	20.09%	0.0550%
Bisignano	Rural	29	10,128	1351	13.34%	0.0365%
Torano C.	Rural	33	4605	813	17.65%	0.0484%
Total			137,912	29,031	21.05%	0.0577%

**Figure A1:**
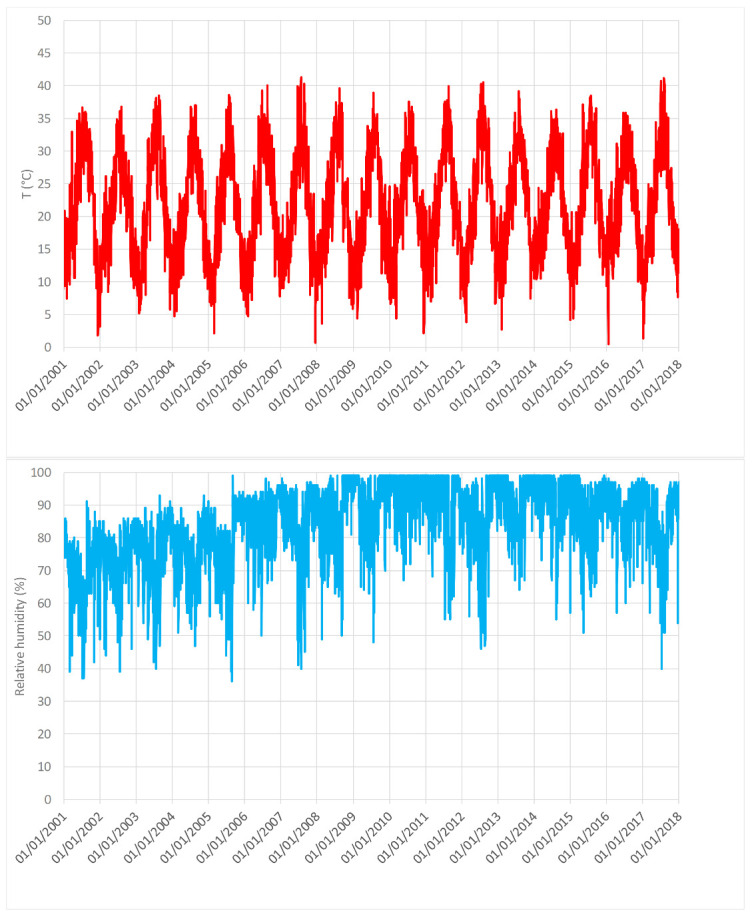
Daily temperature and relative humidity data registered in the Cosenza gauge in the period 2001–2017.

## Data Availability

The data presented in this study are available on request from the corresponding authors.
